# Circulating Soluble Thrombomodulin Is Elevated in Early-Stage CKD and Is Associated with Renal Dysfunction: A Transcriptomic and Retrospective Cohort Study

**DOI:** 10.3390/cells15181639

**Published:** 2026-09-10

**Authors:** Jiao Wang, Yongfen Xiong, Chengyu Liu, Shun Wang, Wenli Wu

**Affiliations:** 1 Department of Clinical Laboratory, Traditional Chinese and Western Medicine Hospital of Wuhan, Tongji Medical College, Huazhong University of Science and Technology, Wuhan 430030, China; wj5289@whyyy.com (J.W.); xyf5161@whyyy.com (Y.X.); 2 Department of Transfusion Medicine, Traditional Chinese and Western Medicine Hospital of Wuhan, Tongji Medical College, Huazhong University of Science and Technology, Wuhan 430030, China; lcy5699@whyyy.com; 3 Central Laboratory, Traditional Chinese and Western Medicine Hospital of Wuhan, Tongji Medical College, Huazhong University of Science and Technology, Wuhan 430030, China

**Keywords:** chronic kidney disease, soluble thrombomodulin, THBD, endothelial injury, renal dysfunction, coagulation, biomarker, renal fibrosis

## Abstract

**Highlights:**

**What are the main findings?**

- THBD is enriched in renal endothelial cells and is consistently upregulated during kidney injury in both bulk and single-cell transcriptomic datasets.
- Circulating sTM is elevated as early as stage 1 CKD, is independently associated with renal dysfunction, and correlates with hemostatic markers.

**What are the implications of the main findings?**

- sTM is a candidate marker of CKD severity reflecting both endothelial injury and reduced renal elimination, pending prospective validation.
- sTM should complement, not replace, conventional renal indices; endothelial dysfunction may link kidney injury, coagulation, and cardiorenal complications.

**Abstract:**

Chronic kidney disease (CKD) is characterized by progressive renal dysfunction and endothelial injury. Thrombomodulin (TM), encoded by the THBD gene, is released into the circulation as soluble TM (sTM) following endothelial damage; however, its clinical significance in CKD remains unclear. To address this question, public bulk and single-cell transcriptomic datasets were analyzed to characterize THBD expression and cellular localization in kidney injury, and a retrospective cohort of 278 hospitalized patients was used to evaluate circulating sTM levels across CKD stages and their associations with renal function, coagulation, and cardiac biomarkers. The transcriptomic analyses showed that THBD was upregulated in injured kidneys, positively correlated with a fibrosis-related transcriptional signature, and predominantly expressed in renal endothelial cells. Consistent with these findings, circulating sTM was elevated as early as stage 1 CKD and increased progressively with advancing disease stage. Higher sTM levels were positively associated with serum creatinine, serum urea, and urinary albumin-to-creatinine ratio (UACR), and inversely associated with estimated glomerular filtration rate (eGFR). In addition, sTM showed weak-to-modest associations with coagulation, fibrinolytic, and cardiac biomarkers. Multivariable analysis showed that eGFR remained independently associated with log-transformed sTM after adjustment for major clinical covariates (*β* = −0.0116, 95% CI −0.0141 to −0.0090, *p* < 0.001). ROC analysis showed that sTM discriminated CKD from non-CKD (AUC = 0.971) and early-stage CKD from non-CKD (AUC = 0.854), outperforming eGFR (AUC 0.920 and 0.565, respectively) and performing comparably to UACR. Collectively, these findings support sTM as a candidate biomarker associated with CKD severity whose interpretation should integrate endothelial injury and reduced renal elimination and prospective validation is warranted.

## 1. Introduction

Chronic kidney disease (CKD) is a progressive disorder defined by persistent abnormalities of kidney structure or function with implications for health. It has become a major global health burden: in 2023, an estimated 788 million adults were living with CKD, and the disease accounted for approximately 1.48 million deaths worldwide. Most affected individuals were classified within CKD stages 1–3, in which symptoms may be absent and opportunities for early intervention are frequently missed [1,2]. Although the causes of CKD are heterogeneous, progressive diseases commonly converge on renal inflammation, microvascular rarefaction, tissue hypoxia, and fibrosis, ultimately leading to irreversible loss of kidney function and kidney failure [3]. Current clinical assessment relies predominantly on serum creatinine-derived estimated glomerular filtration rate (eGFR) and urinary albumin or protein excretion. These indices are indispensable for CKD diagnosis and risk stratification but mainly reflect filtration impairment or established structural damage and do not directly characterize the endothelial and hemostatic abnormalities that may arise during early kidney injury. Identifying biomarkers linked to these pathophysiological processes may therefore improve the biological assessment of CKD and complement conventional renal-function indices.

The vascular endothelium is essential for renal microcirculatory homeostasis, regulation of vascular permeability, inflammatory-cell trafficking, and maintenance of an antithrombotic surface. In CKD, the accumulation of uremic toxins, oxidative stress, chronic inflammation, metabolic abnormalities, and altered shear stress progressively impair protective endothelial functions [4]. Renal endothelial injury can promote capillary loss, impaired tissue perfusion, leukocyte recruitment, and fibrotic remodeling, while systemic endothelial dysfunction contributes to the markedly increased cardiovascular risk associated with CKD. At the same time, patients with CKD may exhibit a complex hemostatic phenotype characterized by activation of coagulation and fibrinolysis, altered platelet function, and coexisting thrombotic and bleeding tendencies [4,5,6,7]. A circulating molecule that reflects endothelial injury while also relating to renal dysfunction and coagulation abnormalities could therefore provide clinically relevant information across the kidney-vascular axis.

Thrombomodulin (TM), also known as CD141 and encoded by the THBD gene, is a multidomain type I transmembrane glycoprotein expressed predominantly on vascular endothelial cells [8]. Its extracellular epidermal growth factor-like domains bind thrombin and redirect thrombin activity toward activation of protein C, thereby suppressing coagulation-factor activation and supporting anticoagulant, anti-inflammatory, cytoprotective, and endothelial-barrier functions. The N-terminal lectin-like domain additionally modulates inflammatory and complement pathways [9]. During endothelial activation or injury, membrane-bound TM can be cleaved and released into the circulation as soluble thrombomodulin (sTM). Circulating sTM is consequently regarded as a marker of endothelial damage, although its concentration may also be influenced by renal elimination and other systemic conditions. This dual dependence is particularly relevant in CKD and requires sTM to be interpreted as an integrated marker rather than a completely endothelial-specific signal.

Experimental and clinical observations support a relationship between the thrombomodulin pathway and kidney injury. Thrombomodulin-dependent activated protein C signaling protects glomerular endothelial cells and podocytes in experimental diabetic nephropathy, whereas loss of the lectin-like domain aggravates complement activation, albuminuria, and glomerular damage [10,11]. Administration of soluble TM also improves renal microvascular perfusion and attenuates inflammation and tissue injury after experimental renal ischemia–reperfusion [12]. In human diabetic nephropathy, glomerular THBD mRNA may increase despite reduced endothelial TM protein, a pattern consistent with compensatory transcription accompanied by protein loss or shedding [13]. Earlier clinical studies reported that circulating TM increases as renal function declines and with greater proteinuria [14,15,16], and subsequent studies have associated higher TM concentrations with CKD stage, renal-function impairment, oxidative stress, and cardiovascular abnormalities [17,18]. However, important questions remain regarding the expression and cellular distribution of THBD across kidney-injury states, whether circulating sTM is altered at an early CKD stage before clear changes in conventional filtration indices, and how sTM relates to global coagulation dynamics, routine coagulation and fibrinolytic markers, and cardiorenal laboratory abnormalities.

Accordingly, the present study integrated public bulk and single-cell transcriptomic datasets with a retrospective clinical analysis. We first examined THBD expression in experimental kidney injury, its association with fibrosis-related transcriptional changes, and its cellular localization in healthy, acute kidney injury, and CKD kidneys. We then evaluated circulating sTM in clinical patients, including its changes in early CKD and across CKD stages, and assessed its relationships with renal-function and urinary albumin excretion. Because TM lies at the interface between endothelial biology and hemostasis, we further explored the associations of sTM with thromboelastography parameters, conventional coagulation and fibrinolytic markers, and cardiac biomarkers. Three aspects distinguish this work from prior reports: (i) it combines multi-level THBD expression analysis—experimental injury models, bulk transcriptomic datasets, and single-cell localization in human kidneys—with a clinical cohort of hospitalized patients; (ii) it evaluates sTM from stage 1 CKD onward, before any significant change in serum creatinine or any decline in eGFR becomes apparent; and (iii) it relates sTM to thromboelastography-derived and conventional coagulation/fibrinolytic parameters, providing a broader hemostatic profile than studies restricted to renal indices alone. We hypothesized that THBD/sTM would be associated with kidney injury and CKD severity and would also reflect broader endothelial-coagulation and cardiorenal disturbances.

## 2. Materials and Methods

### 2.1. Study Design

**Figure 1:**
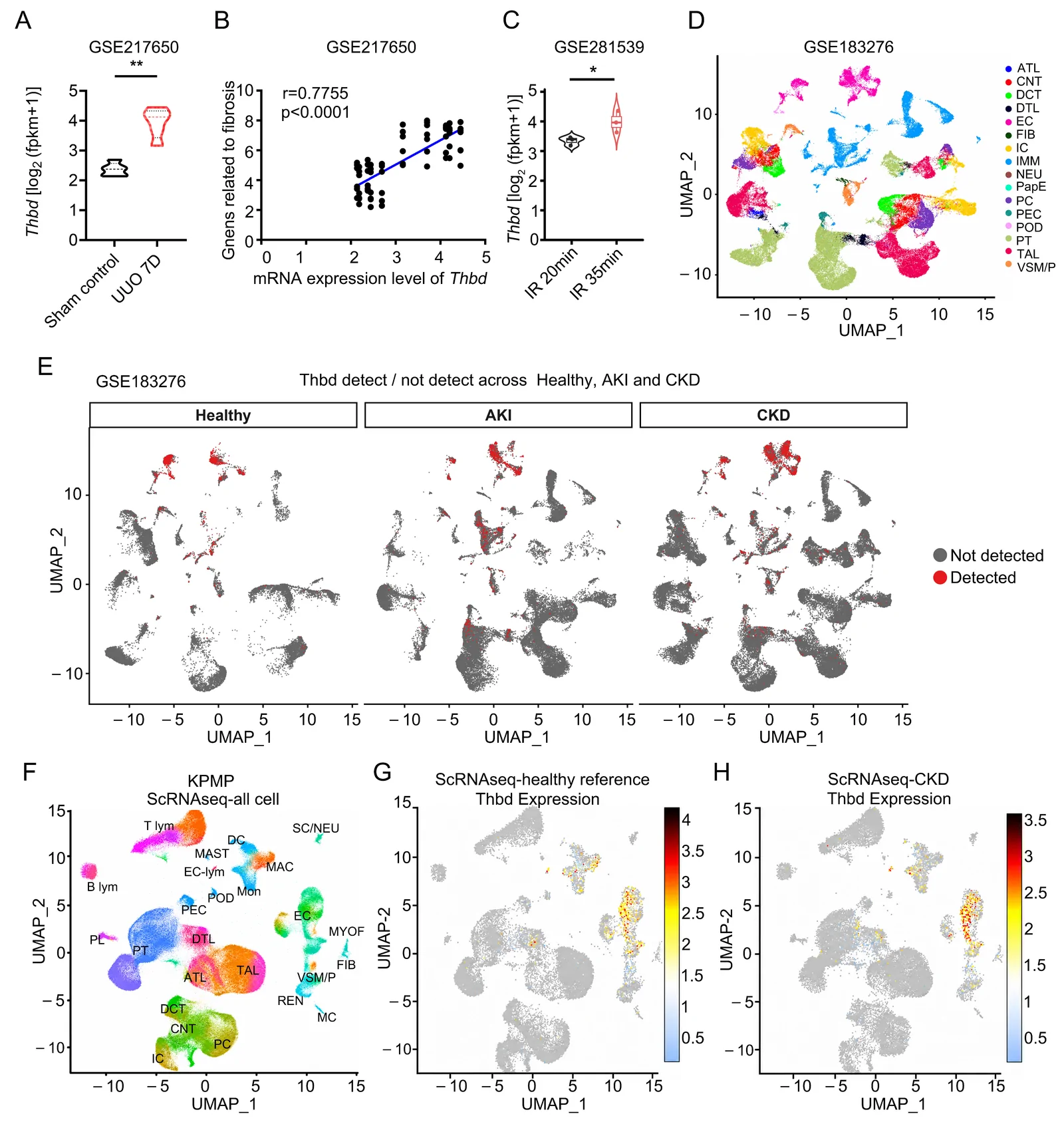
THBD expression is increased in renal injury and is predominantly localized to renal endothelial cells. (**A**) *Thbd* mRNA expression in kidneys from sham-operated mice and mice 7 days after unilateral ureteral obstruction (UUO) in the GSE217650 dataset. Data are shown as mean ± SD. The two groups were compared using Welch’s *t* test (two-tailed). ** *p* < 0.01. (**B**) Pearson correlation between Thbd expression and a renal fibrosis-related gene score in GSE217650. (**C**) *Thbd* mRNA expression in kidney samples from C57BL/6 mice subjected to mild (20 min) or severe (35 min) ischemia–reperfusion injury (IRI) 7 days in the GSE281539 dataset, and was used to investigate transcriptional alterations associated with the AKI-to-CKD transition. The two groups were compared using Welch’s *t* test (two-tailed). * *p* < 0.05. (**D**) Uniform manifold approximation and projection (UMAP) visualization of major renal cell populations in the human kidney single-cell RNA-sequencing dataset GSE183276. (**E**) Distribution of THBD-detected and THBD-undetected cells in healthy, acute kidney injury (AKI), and chronic kidney disease (CKD) samples from GSE183276. (**F**) UMAP visualization of major renal cell populations in the Kidney Precision Medicine Project (KPMP) single-cell RNA-sequencing dataset. (**G**,**H**) Feature plots showing THBD expression in the healthy reference kidney (**G**) and CKD kidney samples (**H**). Color intensity indicates normalized THBD expression. THBD expression was enriched predominantly in renal endothelial cell populations. (Abbreviations: ATL, ascending thin limb; DTL, descending thin limb; PT, proximal tubule; TAL, thick ascending limb; DCT, distal convoluted tubule; CNT, connecting tubule; PC, principal cell; IC, intercalated cell; POD, podocyte; PEC, parietal epithelial cell; EC, endothelial cell; EC-lym: lymphatic endothelial cell; FIB, fibroblast; MYOF, myofibroblast; VSM/P, vascular smooth muscle cell/Pericyte; MC, mesangial cell; REN, renin cell; PapE, papillary epithelial cell; IMM, immune cell; T lym, T lymphocyte; B lym, B lymphocyte; Mon, monocyte; MAC, macrophage; DC, dendritic cell; MAST, mast cell; NEU, neutrophil; SC/NEU, schwann cell/neuron).

**Figure 2:**
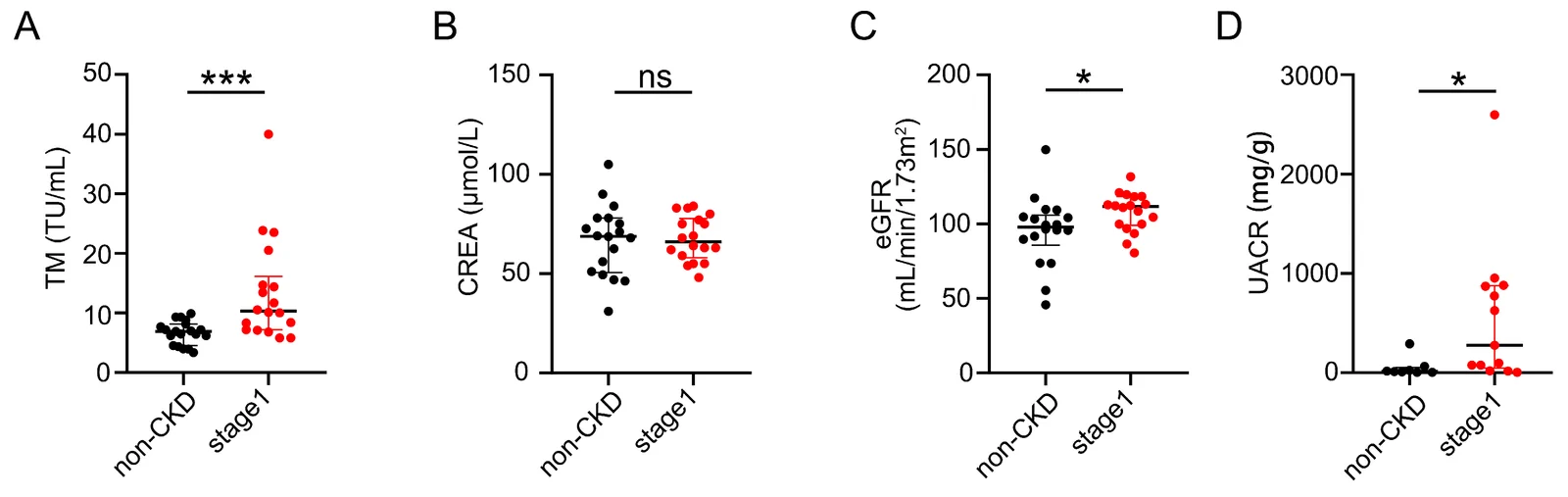
Circulating soluble thrombomodulin is elevated in patients with early-stage CKD. (**A**–**D**) Comparison of circulating soluble thrombomodulin (sTM) concentration (**A**), serum creatinine (CREA) level (**B**), estimated glomerular filtration rate (eGFR) (**C**), and urinary albumin-to-creatinine ratio (UACR) (**D**) between participants without CKD and patients with stage 1 CKD. Individual data points are shown together with the median (IQR). Two-group comparisons were performed using the Mann–Whitney U test (see Section 2.5 for details). * *p* < 0.05; *** *p* < 0.001; ns, not significant. CKD stages were assigned based on longitudinal clinical diagnosis (see Section 2.3).

**Figure 3:**
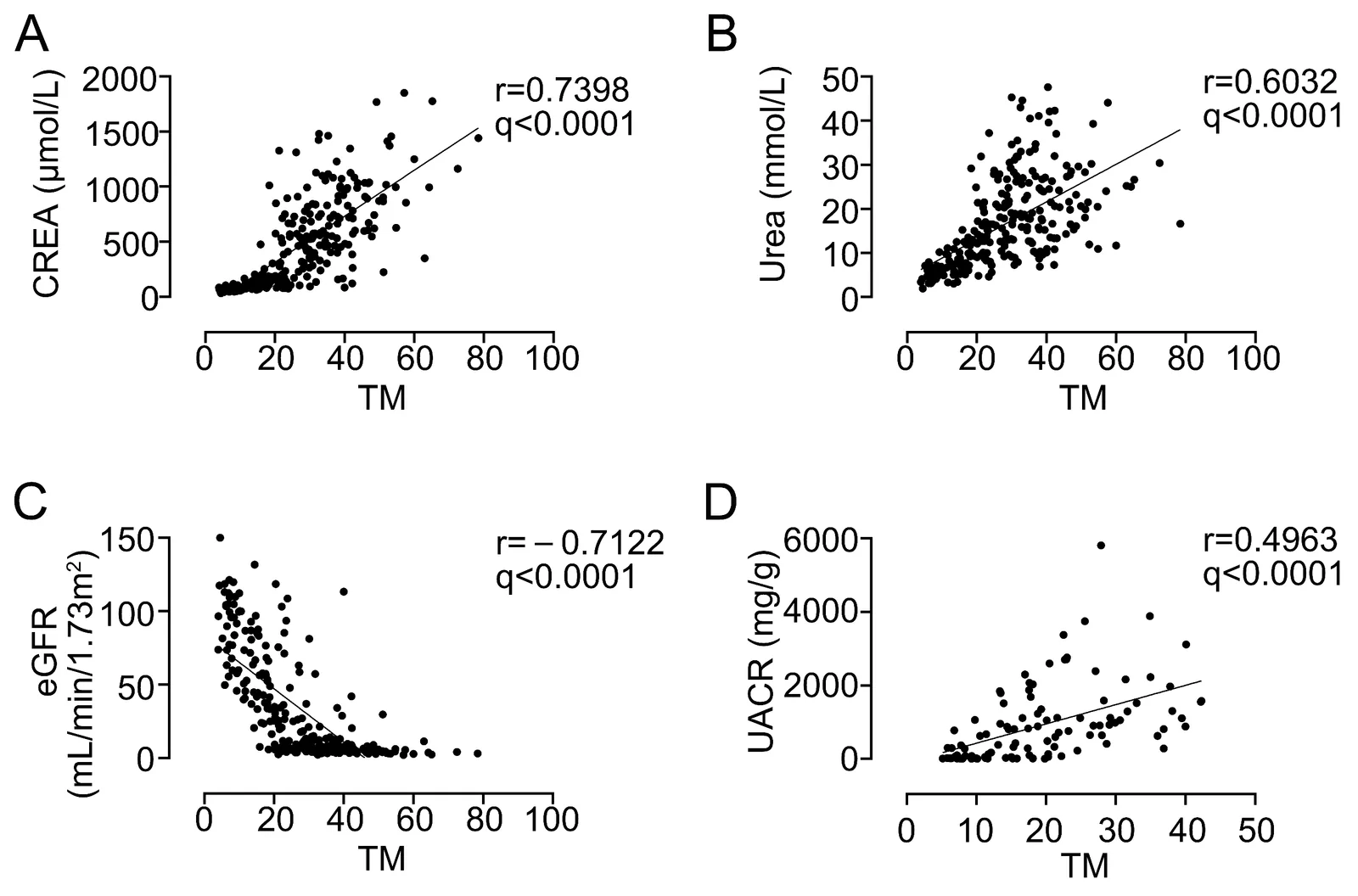
Associations between circulating soluble thrombomodulin and renal function parameters in the study cohort. (**A**–**D**) Pearson correlation analyses between circulating soluble thrombomodulin (sTM) and serum creatinine (CREA) (**A**), serum urea (**B**), estimated glomerular filtration rate (eGFR) (**C**), and urinary albumin-to-creatinine ratio (UACR) (**D**). Given the exploratory nature of the correlation analyses, *p* values were adjusted using the Benjamini–Hochberg false discovery rate (FDR) procedure across 20 tested associations; all panels shown survived FDR correction (*q* < 0.05). Correlation coefficients (r) and corresponding *q* values are shown in each panel.

**Figure 4:**
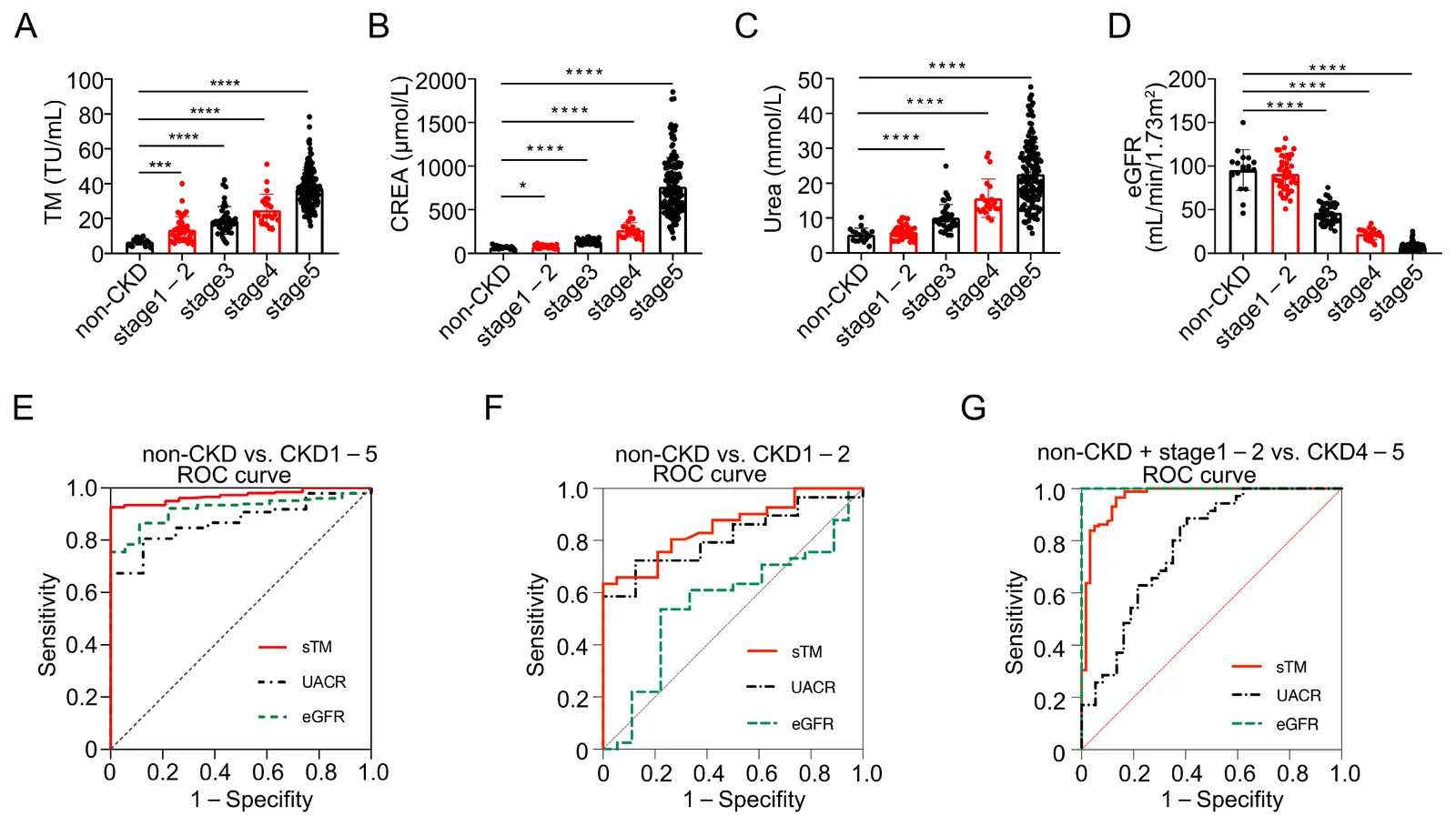
Circulating soluble thrombomodulin increases progressively with CKD severity and discriminates CKD. (**A**–**D**) Comparison of circulating soluble thrombomodulin (sTM) concentration (**A**), serum creatinine (CREA) (**B**), serum urea (**C**), and estimated glomerular filtration rate (eGFR) (**D**) among the non-CKD group and patients with CKD stages 1–2, 3, 4, and 5. Individual data points are shown together with the mean ± SD. Group comparisons in (**A**–**D**) were analyzed by Welch’s ANOVA followed by Dunnett’s T3 post-hoc tests (see Section 2.5); asterisks denote Dunnett’s T3-adjusted *p* values: * *p* < 0.05, *** *p <* 0.001, and **** *p* < 0.0001; (**E**–**G**) Receiver operating characteristic (ROC) curves for sTM, eGFR, and UACR in discriminating CKD, including CKD1-5 from non-CKD (**E**), CKD1-2 from non-CKD (**F**), and CKD4-5 from non-CKD and CKD1-2 combined (**G**). Area under the curve (AUC), confidence interval (CI), optimal cutoffs, sensitivities, specificities, and DeLong test comparisons are reported in Table A1.

**Figure 5:**
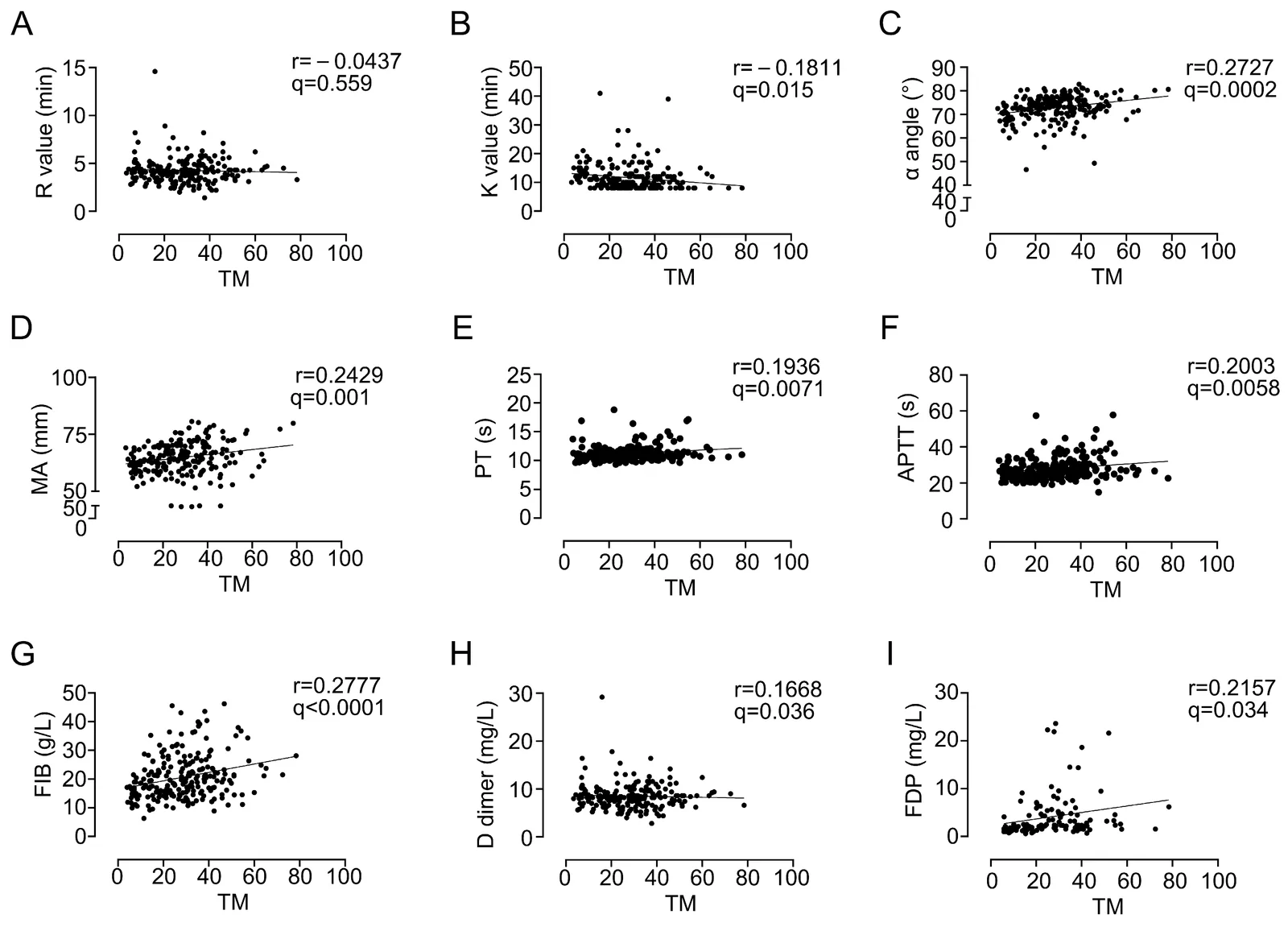
Associations between circulating soluble thrombomodulin and thromboelastography, coagulation, and fibrinolytic parameters in the study cohort. (**A**–**D**) Pearson correlation analyses between circulating soluble thrombomodulin (sTM) and thromboelastography (TEG) parameters, including reaction time (R value) (**A**), clot formation time (K value) (**B**), α angle (**C**), and maximum amplitude (MA) (**D**). (**E**–**I**) Pearson correlation analyses between circulating sTM and conventional coagulation and fibrinolytic parameters, including prothrombin time (PT) (**E**), activated partial thromboplastin time (APTT) (**F**), fibrinogen (FIB) (**G**), D-dimer (**H**), and fibrin(ogen) degradation products (FDP) (**I**). *p* values were adjusted using the Benjamini–Hochberg false discovery rate (FDR) procedure across all 20 correlation tests shown in Figure 3, Figure 5 and Figure 6; R did not survive FDR correction (*q* = 0.559) and is shown for transparency. Correlation coefficients (*r*) and corresponding *q* values are shown in each panel.

**Figure 6:**
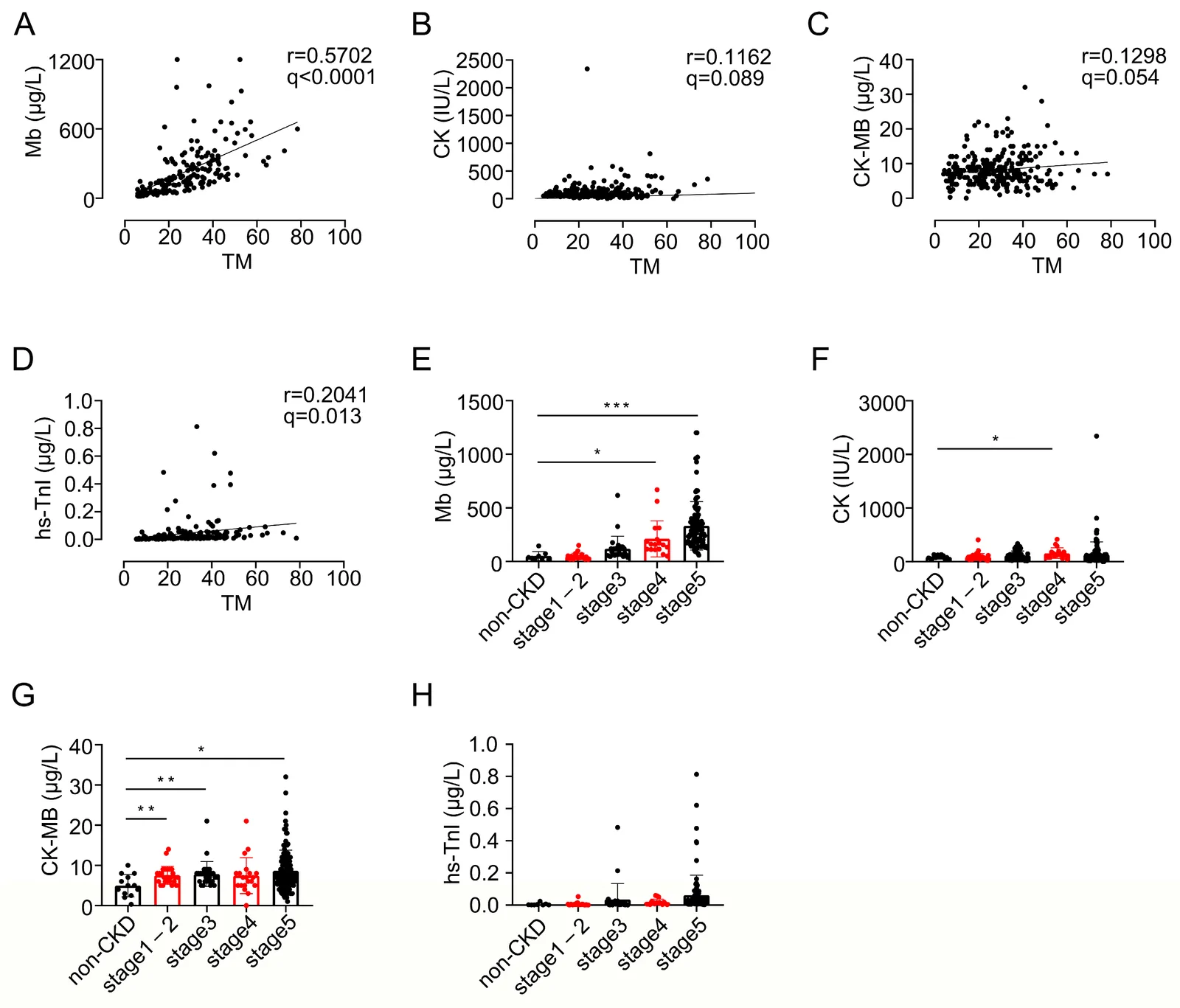
Associations between circulating soluble thrombomodulin and cardiac biomarkers in the study cohort. (**A**–**D**) Pearson correlation analyses between circulating soluble thrombomodulin (sTM) and myoglobin (Mb) (**A**), creatine kinase (CK) (**B**), creatine kinase-MB (CK-MB) (**C**), and high-sensitivity cardiac troponin I (hs-TnI) (**D**). (**E**–**H**) Comparison of serum Mb (**E**), CK (**F**), CK-MB (**G**), and hs-TnI (**H**) levels among the non-CKD group and patients with CKD stages 1–2, 3, 4, and 5. *p* values were adjusted using the Benjamini–Hochberg false discovery rate (FDR) procedure across all 20 correlation tests shown in Figure 3, Figure 5 and Figure 6. Correlation coefficients (*r*) and corresponding *q* values are shown in the correlation plots. Individual data points are presented together with the mean ± SD in the group comparisons. Group comparisons in (**E**–**H**) were analyzed by Welch’s ANOVA followed by Dunnett’s T3 post-hoc tests (see Section 2.5); asterisks denote Dunnett’s T3-adjusted *p* values: * *p* < 0.05, ** *p* < 0.01, *** *p* < 0.001.

This study combined analysis of publicly available transcriptomic datasets with a single-center retrospective analysis of routinely collected clinical data. Figure 1 was generated from public bulk and single-cell RNA-sequencing datasets. Figure 2, Figure 3, Figure 4, Figure 5 and Figure 6, Table 1, Table A1, Table A2, Table A3 and Table A4 were based on clinical data obtained during routine care. No study-specific blood collection, laboratory testing, or intervention was performed.

### 2.2. Public Transcriptomic Data Analysis

Publicly available transcriptomic datasets were obtained from the NCBI Gene Expression Omnibus (GEO) database. GSE217650 comprises bulk RNA-seq data from C57BL/6 mouse kidneys (five controls and five samples collected 7 days after unilateral ureteral obstruction [UUO]) and was used to compare gene expression in renal fibrosis by Welch’s *t* test (two-tailed) [19]. A fibrosis-related expression score was calculated using *Tgfb1*, *Acta2*, *Fn1*, *Col1a1*, *Col3a1*, *Col4a1*, *Vim*, and its association with *Thbd* expression was assessed using Pearson correlation (two-tailed). GSE281539 contains kidney samples from C57BL/6 mice subjected to unilateral nephrectomy combined with mild (20 min) or severe (35 min) ischemia–reperfusion injury (IRI) for 7days, with three samples in each group, and was used to compare transcriptional alterations associated with the AKI-to-CKD transition by Welch’s *t* test (two-tailed) [20]. GSE183276 comprises human kidney single-cell RNA-seq data, including healthy and diseased kidneys (AKI and CKD), and was used to characterize THBD expression across renal cell populations and disease states [21]. Annotated Kidney Precision Medicine Project (KPMP) data were also examined to validate the cellular localization of THBD in healthy and CKD kidneys.

### 2.3. Clinical Participants

Patients admitted to the Department of Nephrology at Wuhan No. 1 Hospital between 1 January and 31 July 2022 who underwent sTM testing during hospitalization were retrospectively screened. For patients with more than one hospitalization during the study period, only the index hospitalization—defined as the first admission at which sTM was measured—was retained; data from readmissions were not analyzed. This ensured that each patient contributed a single observation and that no patient was included more than once. A total of 278 patients were included, comprising 259 patients with CKD and 19 patients who did not meet the study definition of CKD and served as the non-CKD comparison group. The non-CKD group was not considered a healthy population.

CKD was diagnosed in accordance with the KDIGO 2024 Clinical Practice Guideline for the Evaluation and Management of Chronic Kidney Disease [2], which defines CKD as abnormalities of kidney structure or function, present for a minimum of 3 months, with implications for health. Given the retrospective design of this study, the diagnosis and staging of CKD were based on the clinical judgment of the treating physicians as documented in the medical records, which integrated laboratory findings obtained longitudinally over the patients’ clinical course-including serial eGFR and urinary albumin measurements-rather than on a single assessment. CKD stages 1–5 were assigned according to the GFR categories (G1–G5) of the KDIGO classification. Because within-patient eGFR may fluctuate over time, the eGFR values analyzed in this study, which were derived from measurements obtained during the index hospitalization, may not be fully concordant with the assigned CKD stage in individual patients.

### 2.4. Clinical Data Collection

Demographic characteristics, comorbidities, dialysis status, and laboratory results were extracted from the electronic medical record and laboratory information system. Variables included renal indices (serum urea, CREA, eGFR, and UACR), sTM, thromboelastography parameters (R, K, α angle, and MA), conventional coagulation and fibrinolytic indices (PT, APTT, fibrinogen, D-dimer, FDP, TAT, PIC, and tPAIC), and cardiac biomarkers (Mb, CK, CK-MB, and hs-TnI). Measurement of sTM was performed on the Sysmex HISCL-5000 platform using the manufacturer’s matching reagents. Additional demographic, inflammatory, and metabolic variables used in Table 1 were collected from the same records, and the eGFR was calculated using the 2021 CKD-EPI creatinine equation based on serum creatinine level.

### 2.5. Statistical Analysis

Continuous variables were expressed as mean ± standard deviation (SD) and categorical variables as numbers and percentages. Non-normally distributed continuous variables are additionally presented as median (IQR) where reported (e.g., Figure 2).

Continuous variables were compared across the five study groups (the non-CKD group and patients with CKD stages 1–2, 3, 4, and 5) using Welch’s ANOVA, which was adopted because group variances were unequal (Levene’s test), followed by Dunnett’s T3 post-hoc tests for all pairwise comparisons. This procedure was applied to all group-wise comparisons of continuous variables, namely those summarized in Table 1 and those presented in Figure 4A–D and Figure 6E–H. Because Dunnett’s T3 procedure controls the family-wise error rate across all pairwise comparisons within each variable, no additional multiple-comparison correction was applied; the resulting adjusted *p* values are indicated by superscript letters in Table 1 and by asterisks in the figures.

Because sTM and UACR violated the normality assumption (Shapiro–Wilk *p* < 0.05) and UACR showed unequal variances (Levene’s test *p* = 0.046), the Mann–Whitney U test was applied to all four parameters for internal consistency.

Associations between sTM and renal indices, thromboelastography parameters, coagulation and fibrinolytic variables, and cardiac biomarkers were assessed using Pearson correlation, with two-sided *p* values. Because these correlation analyses were exploratory and comprised 20 comparisons, *p* values were adjusted using the Benjamini–Hochberg false discovery rate (FDR) procedure; *q* < 0.05 was considered significant.

To assess incremental diagnostic performance, receiver operating characteristic (ROC) curves were constructed and the area under the curve (AUC) was computed for sTM, eGFR (with the sign reversed), and UACR to discriminate (i) CKD (all stages) versus non-CKD, (ii) early-stage CKD (stages 1–2) versus non-CKD, and (iii) advanced CKD (stages 4–5) versus non-CKD plus stages 1–2. AUCs were compared using the DeLong test, and 95% confidence intervals (CI) were calculated by the DeLong variance method. Optimal cutoffs were selected by the Youden index, and sensitivity and specificity were reported. In sensitivity analyses, all dialysis patients were excluded (*n* = 149); sTM levels were re-compared across CKD stages using the Kruskal–Wallis test (Table A2), and Pearson correlations with renal indices were re-computed, to verify that the stage-dependent sTM elevation and its associations with renal function were not driven by dialysis status.

To evaluate whether the association between sTM and renal function was independent of demographic characteristics and comorbidities, multivariable linear regression was performed with log-transformed sTM (natural logarithm) as the dependent variable and eGFR as the primary predictor: Model 1, eGFR only; Model 2, eGFR + age + sex; Model 3, Model 2 + hypertension + diabetes mellitus + heart failure + dialysis + log-transformed UACR; Model 4, Model 2 + hypertension + diabetes mellitus + heart failure + dialysis + log-transformed C-reactive protein. Models were fitted on complete cases; because UACR and C-reactive protein were not measured in all patients, the analytic sample sizes differed across models (Table A3).

Statistical analyses were performed using Python 3.13 (SciPy, NumPy, pandas) and GraphPad Prism 9 (GraphPad Software, San Diego, CA, USA). All tests were two-sided, and *p* < 0.05 was considered statistically significant.

## 3. Results

### 3.1. Transcriptomic Discovery Identifies THBD Induction and Endothelial Enrichment in Kidney Injury

To investigate the potential of THBD as a kidney injury biomarker, we first analyzed publicly available bulk and single-cell transcriptomic datasets. In the murine unilateral ureteral obstruction (UUO) dataset GSE217650, Thbd mRNA expression was significantly higher in obstructed kidneys than in sham-operated controls (Figure 1A). Thbd expression was also strongly and positively correlated with a fibrosis-related gene signature (*r* = 0.7755, *p* < 0.0001; Figure 1B), linking its induction to renal fibrotic remodeling. In the murine unilateral nephrectomy combined with ischemia–reperfusion injury (IRI) 7 days dataset GSE281539, Thbd mRNA expression was significantly higher in severe (35 min) IRI kidneys than in mild (20 min) (Figure 1C).

We next examined the cellular distribution of THBD in the human kidney single-cell RNA-sequencing dataset GSE183276. UMAP-based clustering identified the major epithelial, endothelial, stromal, and immune cell populations (Figure 1D). THBD-positive cells were detectable in healthy, acute kidney injury (AKI), and chronic kidney disease (CKD) samples, with a broader distribution of THBD-positive cells in the injured kidneys (Figure 1E). Analysis of the Kidney Precision Medicine Project dataset further showed that THBD expression was concentrated predominantly in endothelial cell populations in both the healthy reference and CKD kidneys (Figure 1F–H). These findings identify THBD as an injury-associated, endothelial-enriched molecule and provided supportive, hypothesis-generating evidence for evaluating circulating sTM in the clinical cohort. They do not establish a direct quantitative relationship between tissue THBD transcription and circulating sTM concentrations in the same individuals.

### 3.2. Circulating sTM Is Elevated in Early-Stage CKD Despite Unchanged Serum Creatinine

To translate these transcriptomic observations into a clinical context, we first compared circulating sTM levels between non-CKD controls and patients with stage 1 CKD. Serum sTM concentrations were significantly higher in the stage 1 CKD group (*p* < 0.001; Figure 2A), whereas serum CREA did not differ significantly (Figure 2B), and eGFR and UACR were significantly higher in stage 1 CKD (*p* < 0.05; Figure 2C,D). Thus, an increase in circulating sTM was detectable at an early stage of CKD when changes in CREA were not yet apparent, while eGFR and albuminuria already showed significant differences.

### 3.3. Clinical Characteristics of the Study Cohort and Stage-Dependent Elevation of sTM

A total of 278 patients admitted to the Department of Nephrology at Wuhan No. 1 Hospital between 1 January and 31 July 2022 and tested for sTM during hospitalization were included in the study; each patient contributed a single hospitalization. Patients were categorized as non-CKD, CKD stages 1–2, stage 3, stage 4, or stage 5 according to the prespecified clinical classification. Their demographic characteristics, comorbidities, renal indices, coagulation variables, inflammatory markers, and other laboratory measurements are summarized in Table 1.

### 3.4. Circulating sTM Is Closely Associated with the Severity of Renal Dysfunction

We next quantified the association between sTM and established measures of kidney function. Circulating sTM was strongly positively correlated with CREA (*r* = 0.7398, *q* < 0.0001; Figure 3A) and urea (*r* = 0.6033, *q* < 0.0001; Figure 3B), and strongly negatively correlated with eGFR (*r* = −0.7122, *q* < 0.0001; Figure 3C). A moderate positive correlation was also observed between sTM and UACR (*r* = 0.4963, *q* < 0.0001; Figure 3D). These findings associate higher circulating sTM with both impaired filtration and greater urinary albumin excretion.

Group-wise analyses provided complementary evidence of this relationship. sTM increased progressively across CKD stages and was already significantly elevated in stages 1–2 relative to the non-CKD group (Figure 4A). CREA and urea increased predominantly with more advanced disease (Figure 4B,C), whereas eGFR progressively declined (Figure 4D). The early elevation and subsequent stage-dependent accumulation of sTM therefore support its potential as a marker of CKD presence and severity.

To evaluate its incremental diagnostic performance beyond established renal measures, we performed dedicated receiver operating characteristic and multivariable analyses. Receiver operating characteristic analysis was used to evaluate the incremental diagnostic performance of sTM relative to eGFR and urinary albumin-to-creatinine ratio (UACR). For distinguishing CKD (all stages) from non-CKD, sTM yielded an AUC of 0.971, 95% CI 0.952–0.989 at an optimal cutoff of 10.0 TU/mL (sensitivity 92.7%, specificity 100%), which was higher than eGFR (AUC 0.920, 95% CI 0.880–0.959; DeLong test, *p* = 0.0084) but not statistically different from UACR (AUC 0.872, 95% CI 0.787–0.958; *p* = 0.3726). In the early-stage CKD (stages 1–2) versus non-CKD comparison, sTM achieved an AUC of 0.854, 95% CI 0.762–0.947 at the same cutoff, significantly outperforming eGFR (AUC 0.565, 95% CI 0.403–0.727; *p* = 0.0012) and comparable to UACR (AUC 0.815, 95% CI 0.674–0.955; *p* = 0.7082). For advanced CKD (stages 4–5) versus non-CKD plus stages 1–2, sTM also performed well (AUC 0.967, 95% CI 0.940–0.995, cutoff 18.4 TU/mL, sensitivity 96.6%, specificity 86.7%), and comparably to UACR (AUC 0.967 vs. 0.781, *p* < 0.001). But eGFR outperformed sTM (AUC 1.000 vs. 0.967, *p* = 0.0272), consistent with eGFR directly reflecting stage-defining filtration thresholds; the incremental value of sTM was most evident in early-stage CKD. These findings suggest that sTM may offer incremental diagnostic information, particularly for early CKD detection, in this cohort. Full ROC results and DeLong comparisons are provided in Figure 4E–G and Table A1.

In sensitivity analyses excluding dialysis patients, sTM remained progressively elevated across CKD stages (Kruskal–Wallis *H* = 87.9, *p* < 0.0001; Table A2) and correlated strongly with CREA (*r* = 0.664, *p* < 0.0001), urea (*r* = 0.687, *p* < 0.0001), eGFR (*r* = −0.666, *p* < 0.0001), and UACR (*r* = 0.529, *p* < 0.0001). These results indicate that the observed associations were not driven by dialysis status.

Multivariable analyses were performed with log-transformed sTM as the dependent variable to determine whether the association between sTM and renal dysfunction was independent of eGFR and comorbidities. In the fully adjusted model (*n* = 105), eGFR remained independently associated with sTM (*β* = −0.0116, 95% CI −0.0141 to −0.0090, *p* < 0.001), indicating that a 10 mL/min/1.73 m^2^ decrease in eGFR corresponded to approximately 12% higher sTM concentration. Proteinuria (log-transformed UACR) was also independently associated with sTM (*β* = 0.0773, 95% CI 0.0416 to 0.1130, *p* < 0.001). These results indicate that the elevation of sTM in CKD cannot be fully explained by reduced glomerular filtration alone, although eGFR remained the strongest determinant. Full model details, including Models 1–4, are provided in Table A3.

### 3.5. Circulating sTM Is Associated with Alterations in Coagulation and Fibrinolytic Parameters

Because thrombomodulin is closely linked to endothelial anticoagulant function, we examined the associations between circulating sTM and both thromboelastography (TEG)-derived and conventional coagulation parameters in an exploratory manner. After Benjamini–Hochberg FDR correction across 20 correlation tests, sTM was not correlated with R (*r* = −0.0437, *q* = 0.559), but showed a weak inverse correlation with clot formation time (K; *r* = −0.1811, *q* = 0.015) and weak positive correlations with the α angle (*r* = 0.2727, *q* < 0.001) and maximum amplitude (MA; *r* = 0.2429, *q* = 0.0010) (Figure 5A–D). In addition, sTM showed significant positive correlations with PT (*r* = 0.1936, *q* = 0.0071), APTT (*r* = 0.2003, *q* = 0.0058), FIB (*r* = 0.2777, *q* < 0.0001), D-dimer (*r* = 0.1668, *q* = 0.036), and FDP (*r* = 0.2157, *q* = 0.034) (Figure 5E–I). Although the effect sizes were modest, the consistent associations across TEG and routine coagulation assays indicate that elevated sTM is accompanied by alterations in clot propagation, clot strength, and fibrinolytic status, supporting a clinical relationship between endothelial injury and hemostatic dysfunction in CKD. Moreover, TAT, PIC, and tPAIC were also examined but were not significantly associated with sTM after FDR correction (Table A4).

### 3.6. Circulating sTM Is Associated with Cardiac-Related Laboratory Biomarkers

Having established associations with renal dysfunction and hemostatic abnormalities, we next explored whether sTM was also related to cardiac-associated laboratory biomarkers. sTM was moderately positively correlated with Mb (*r* = 0.5702, *q* < 0.0001; Figure 6A), CK did not survive FDR correction (*r* = 0.1162, *q* = 0.089), and CK-MB showed only a borderline association (*r* = 0.1298, *q* = 0.054) (Figure 6B,C); sTM was weakly but significantly correlated with hs-TnI (*r* = 0.2041, *q* = 0.013; Figure 6D). Mb increased markedly across advanced CKD stages (Figure 6E), whereas CK, CK-MB, and hs-TnI showed less consistent stage-dependent patterns (Figure 6F–H). These exploratory findings suggest that elevated sTM may accompany broader systemic or cardiorenal abnormalities in CKD. However, because these biomarkers can be influenced by reduced renal clearance and coexisting cardiovascular disease, the data do not establish sTM as a cardiac-specific injury marker.

### 3.7. Integrated Summary of the Clinical and Transcriptomic Findings

Collectively, these results provide complementary, hypothesis-supporting evidence linking transcriptomic observations to clinical findings. THBD was induced in injured kidneys and predominantly enriched in renal endothelial cells, whereas circulating sTM was elevated from the early stages of CKD and increased progressively with disease severity. Higher sTM levels were closely associated with impaired renal function and albuminuria and were additionally accompanied by weak-to-moderate alterations in coagulation, fibrinolytic, and cardiac-associated laboratory parameters. These results support circulating sTM as a candidate biomarker of CKD progression whose interpretation should consider both endothelial injury and reduced renal elimination, while suggesting its potential relevance to systemic endothelial dysfunction and cardiorenal involvement.

## 4. Discussion

In this study, we combined public transcriptomic datasets with a retrospective clinical cohort to investigate the significance of thrombomodulin in chronic kidney disease (CKD). THBD was upregulated in injured kidneys, positively associated with a fibrosis-related transcriptional signature, and localized predominantly to renal endothelial cells (Figure 1A–H). Clinically, circulating soluble thrombomodulin (sTM) was elevated in early CKD, increased progressively across CKD stages, and was strongly associated with serum creatinine (CREA), serum urea, estimated glomerular filtration rate (eGFR), and moderately associated with UACR (Figure 2, Figure 3 and Figure 4 and Table 1). sTM also showed weak-to-moderate associations with thromboelastographic, coagulation, fibrinolytic, and cardiac-associated laboratory parameters after FDR correction (Figure 5 and Figure 6). These findings support sTM as a candidate marker of CKD severity, while indicating that its elevation occurs in the broader setting of systemic endothelial and hemostatic disturbance.

Our work extends prior clinical reports in three respects. First, although associations between circulating thrombomodulin and worsening renal function have been described (e.g., Guo et al., BMC Nephrol 2025 [18]), most prior cohorts did not evaluate sTM from stage 1 CKD or compare its discriminative performance directly with eGFR and UACR using ROC analyses. Second, few studies have integrated sTM with a comprehensive thromboelastography-derived hemostatic profile alongside conventional coagulation and fibrinolytic markers. Third, the combination of single-cell THBD localization in human kidney datasets with a clinical cohort of hospitalized patients provides a more structured rationale for evaluating circulating sTM than either component alone.

Thrombomodulin is an endothelial transmembrane glycoprotein that binds thrombin and promotes activation of the protein C pathway, thereby exerting anticoagulant, anti-inflammatory, cytoprotective, and barrier-stabilizing effects [9,22]. Endothelial injury can cause proteolytic shedding of membrane thrombomodulin and release of its extracellular domain into the circulation. Circulating sTM is therefore commonly considered an indicator of endothelial damage. Experimental soluble thrombomodulin has been shown to improve renal microvascular perfusion and reduce inflammation and tissue injury in ischemic kidney models, supporting a functional relationship between the thrombomodulin pathway and renal vascular integrity [12].

The transcriptomic findings provide a cellular basis for interpreting circulating sTM in CKD. In the UUO dataset, Thbd expression was increased and correlated with fibrosis-related genes (Figure 1A,B), in the IRI dataset, Thbd expression was also increased and correlated with AKI-to-CKD progression (Figure 1C), whereas single-cell analyses showed that THBD was concentrated mainly in renal endothelial populations (Figure 1D–H). These observations are consistent with the established endothelial expression of THBD and suggest that altered THBD expression is linked to the injured renal vascular microenvironment. However, tissue THBD transcription, endothelial membrane protein abundance, and circulating sTM are not equivalent measures. Increased transcription may represent a compensatory response, whereas elevated circulating sTM may result from enhanced endothelial shedding, reduced renal elimination, or both. Human diabetic nephropathy studies have similarly reported discordance between THBD mRNA and endothelial protein expression [13]. We therefore regard the transcriptomic component as hypothesis-supporting rather than confirmatory.

The clinical data further demonstrated that sTM was already elevated in early-stage CKD, despite no significant group-level differences in CREA in the initial comparison (Figure 2A,B). Notably, UACR was also higher in stage 1 CKD (Figure 2D), indicating that albuminuria was already detectable at this early point. This finding raises the possibility that endothelial injury or altered thrombomodulin turnover may become detectable alongside proteinuria before a marked decline in conventional CREA indices. In the larger cohort, sTM increased progressively from non-CKD to CKD stage 5 (Table 1 and Figure 4A) and correlated strongly with CREA, urea, and eGFR and moderately with UACR (Figure 3A–D). These results are consistent with previous studies showing that circulating thrombomodulin rises with worsening renal function and proteinuria [14,15,16,17,18,23,24]. Thus, sTM may provide information complementary to conventional markers of renal function and kidney damage, rather than replacing them.

An important limitation in interpreting these associations is that circulating sTM is influenced by renal clearance. Previous clinical and pharmacokinetic studies have shown that plasma thrombomodulin concentrations increase as renal function declines and that clearance of recombinant soluble thrombomodulin is reduced in renal impairment [14,23,25]. Therefore, the strong relationships between sTM, CREA, and eGFR in our cohort (Figure 3A–C) may reflect both increased endothelial release and reduced renal elimination. This dual contribution does not eliminate the potential biomarker value of sTM, but it prevents attribution of elevated concentrations solely to endothelial injury. Multivariable linear regression indicated that eGFR was independently associated with log-transformed sTM after adjustment for age, sex, hypertension, diabetes mellitus, heart failure, dialysis, and proteinuria (Table A3), with renal function remaining the dominant determinant of circulating sTM in every model. Notably, proteinuria (log-transformed UACR) was independently associated with sTM even after adjustment for eGFR (Table A3). Because albuminuria reflects glomerular and renal microvascular damage rather than impaired solute clearance, this finding suggests that elevated sTM in CKD is not entirely explained by reduced renal elimination, but also mirrors the severity of kidney injury itself.

The associations between sTM and hemostatic parameters were modest, and only a subset survived FDR correction. CKD is characterized by a complex coagulation phenotype in which thrombotic risk, platelet dysfunction, and bleeding susceptibility may coexist [5,7,26]. In our cohort, sTM was not associated with the thromboelastography reaction time but showed FDR-significant associations with K, the α angle, and maximum amplitude (Figure 5A–D). It was also FDR-significantly correlated with PT, APTT, fibrinogen, D-dimer, and FDP (Figure 5E–I). These findings suggest that elevated sTM occurs alongside alterations in clot formation and fibrin turnover. However, the small effect sizes do not support a direct causal role for sTM in hypercoagulability. Instead, the observed pattern likely reflects shared effects of endothelial injury, inflammation, uremic toxins, and advanced kidney dysfunction on the hemostatic system [7,27].

The relationships between sTM and cardiac-associated biomarkers were exploratory. sTM showed its strongest association with myoglobin, a weaker but FDR-significant correlation with hs-TnI, no significant association with CK, and a borderline association with CK-MB (Figure 6A–D). Because CKD is strongly associated with cardiovascular disease and systemic endothelial dysfunction, these findings may indicate broader cardiorenal involvement [4]. Nevertheless, myoglobin and cardiac troponins may accumulate as renal function declines, and their concentrations are also influenced by skeletal muscle injury, heart failure, dialysis, infection, and other comorbidities [28,29]. Figure 6 should therefore not be interpreted as evidence that sTM is a specific marker of myocardial injury.

From a clinical perspective, the early elevation of sTM and its progressive increase across CKD stages suggest that it may complement established renal biomarkers when assessing endothelial injury and disease severity. However, the present study demonstrates associations rather than causality; the ROC analyses suggest that sTM has higher discriminative ability than eGFR and comparable ability to UACR for detecting early-stage CKD in this cohort (AUC 0.854 vs. 0.565 and 0.815, respectively), but these findings require external validation before claiming incremental diagnostic value. Prospective multicenter studies should compare sTM with CREA, eGFR, UACR, and other endothelial markers, evaluate its independent contribution in multivariable models, and determine whether serial changes predict CKD progression or cardiovascular outcomes.

This study has several limitations. First, it was retrospective, cross-sectional, and conducted at a single center, which may introduce selection bias and limit generalizability. Second, the design does not permit causal inference or evaluation of long-term outcomes. Third, residual confounding from age, comorbidities, inflammation, dialysis status, medication exposure, and CKD etiology is likely. In addition, CKD staging was based on the treating physicians’ longitudinal clinical diagnoses documented in the medical records, rather than on standardized repeated eGFR assessments; therefore, the single eGFR values obtained during the index hospitalization may not be fully concordant with the assigned CKD stage in individual patients. Fourth, reduced renal clearance may contribute substantially to elevated sTM, particularly in advanced CKD. Finally, the coagulation and cardiac-biomarker analyses were exploratory, and we applied Benjamini–Hochberg FDR correction across 20 correlation comparisons; only associations surviving FDR correction should be interpreted with confidence. Transcriptomic expression, membrane thrombomodulin, and circulating sTM were not measured in the same individuals.

In conclusion, our integrated transcriptomic and clinical analyses identify thrombomodulin as a molecule associated with renal dysfunction and CKD severity. THBD was upregulated in injured kidneys and predominantly localized to renal endothelial cells (Figure 1A–H), while circulating sTM was elevated in early CKD and increased progressively with disease stage (Figure 2A and Figure 4A, Table 1). sTM was strongly associated with renal dysfunction and albuminuria (Figure 3A–D) and showed FDR-corrected associations with selected coagulation, fibrinolytic, and cardiac-associated markers (Figure 5 and Figure 6). Multivariable and ROC analyses further indicate that the association of sTM with kidney function persists after adjustment and that sTM may offer discriminative information for early CKD detection in this cohort (Figure 4E–G, Table A1 and Table A3). These findings support further evaluation of circulating sTM as a candidate biomarker of CKD progression.

## 5. Conclusions

THBD is predominantly expressed in renal endothelial cells and is upregulated in injured kidneys. Circulating soluble thrombomodulin is elevated during early CKD, increases progressively with disease severity, and is associated with renal dysfunction as well as selected systemic coagulation and cardiac-related laboratory abnormalities. Multivariable adjustment indicates that eGFR remains independently associated with sTM after accounting for age, sex, comorbidities, dialysis, and proteinuria, and ROC analyses indicate that sTM discriminates CKD and early-stage CKD from non-CKD, outperforming eGFR and performing comparably to UACR in this cohort. These findings support sTM as a promising candidate biomarker reflecting CKD progression and systemic endothelial disturbance, while emphasizing that its interpretation should consider both endothelial damage and renal elimination, although prospective multicenter validation remains warranted.

## Figures and Tables

**Table 1:** Clinical characteristics and laboratory parameters of the study population according to CKD stage.

Stage	Overall	Non-CKD	CKD1–2	CKD3	CKD4	CKD5
Age (years), mean ± SD	61.2 ± 13.3	57.8 ± 13.8	52.7 ± 13.7	66.1 ± 10.2	63.5 ± 13.0	62.2 ± 13.0
Male sex (%)	51.4	26.3	48.8	50.0	56.5	55.0
Hypertension (%)	82.0	57.9	56.1	86.4	95.7	88.7
Diabetes mellitus (%)	34.2	15.8	24.4	45.5	39.1	35.1
Heart failure (%)	23.4	10.5	4.9	20.5	8.7	33.1
Pulmonary infection (%)	26.6	26.3	22.0	29.5	30.4	26.5
Dialysis (%)	46.4	0	0	0	0	85.4
Serum urea (mmol/L), mean ± SD	16.3 ± 10.1	5.18 ± 2.0	6.0 ± 1.9 (ns)	10.1 ± 3.8 (*, a)	15.7 ± 5.5 (*, a, b)	22.6 ± 9.2 (*, a, b, c)
Serum CREA (μmol/L), mean ± SD	469.4 ± 404.9	66.8 ± 17.9	78.4 ± 14.8 (ns)	133.8 ± 29.4 (*, a)	266.0 ± 85.5 (*, a, b)	764.7 ± 334.5 (*, a, b, c)
eGFR (mL/min/1.73 m^2^), mean ± SD	33.6 ± 36.4	95.3 ± 23.2	90.9 ± 19.6 (ns)	46.3 ± 11.9 (*, a)	21.9 ± 6.1 (*, a, b)	6.9 ± 3.7 (*, a, b, c)
UACR (mg/g), mean ± SD	915.38 ± 1038.49	53.47 ± 97.79	719.23 ± 804.54 (*)	700.66 ± 779.09 (*, ns)	975.17 ± 773.00 (*, ns, ns)	1864.72 ± 1449.99 (*, a, b, ns)
sTM (TU/mL), mean ± SD	27.6 ± 14.5	6.7 ± 2.0	13.7 ± 7.4 (*)	18.9 ± 8.0 (*, a)	24.9 ± 9.0 (*, a, b)	36.9 ± 11.0 (*, a, b, c)
TAT (ng/mL), mean ± SD	6.1 ± 10.8	3.3 ± 3.2	8.0 ± 19.3 (ns)	5.9 ± 8.4 (ns, ns)	6.3 ± 8.9 (ns, ns, ns)	6.0 ± 9.1 (ns, ns, ns, ns)
PIC (μg/mL), mean ± SD	1.0 ± 1.1	0.5 ± 0.2	1.3 ± 2.5 (ns)	1.0 ± 0.8 (*, ns)	1.1 ± 1.0 (ns, ns, ns)	1.0 ± 0.6 (*, ns, ns, ns)
tPAIC (ng/mL), mean ± SD	5.8 ± 3.9	5.7 ± 2.1	6.7 ± 3.0 (ns)	7.7 ± 4.6 (ns, ns)	4.8 ± 2.9 (ns, ns, b)	5.3 ± 3.9 (ns, ns, b, ns)
PT (s), mean ± SD	11.4 ± 3.2	11.0 ± 1.2	10.7 ± 1.4 (ns)	10.7 ± 0.7 (ns, ns)	11.0 ± 0.9 (ns, ns, ns)	11.9 ± 4.1 (ns, ns, b, ns)
APTT (s), mean ± SD	28.8 ± 12.7	27.3 ± 3.2	26.1 ± 5.0 (ns)	25.7 ± 6.9 (ns, ns)	26.1 ± 4.7 (ns, ns, ns)	30.8 ± 16.0 (ns, ns, ns, ns)
D-dimer (mg/L), mean ± SD	2.0 ± 3.2	1.2 ± 2.0	0.9 ± 0.9 (ns)	1.8 ± 3.4 (ns, ns)	3.6 ± 7.0 (ns, ns, ns)	2.2 ± 2.7 (ns, a, ns, ns)
FIB (g/L), mean ± SD	4.1 ± 1.5	2.9 ± 0.6	3.9 ± 1.6 (*)	4.1 ± 1.2 (*, ns)	4.1 ± 1.5 (*, ns, ns)	4.3 ± 1.6 (*, ns, ns, ns)
FDP (mg/L), mean ± SD	5.2 ± 9.5	1.3 ± 0.2	2.0 ± 1.5 (ns)	3.0 ± 2.2 (*, ns)	16.4 ± 26.6 (ns, ns, ns)	5.6 ± 5.9 (*, a, b, ns)
R (min), mean ± SD	4.26 ± 1.35	4.41 ± 0.86	4.52 ± 1.38 (ns)	4.05 ± 1.26 (ns, ns)	3.85 ± 0.59 (ns, ns, ns)	4.31 ± 1.47 (ns, ns, ns, ns)
MA (mm), mean ± SD	64.73 ± 6.56	62.21 ± 4.16	62.74 ± 4.77 (ns)	63.43 ± 5.10 (ns, ns)	65.46 ± 5.92 (ns, ns, ns)	65.57 ± 7.29 (ns, ns, ns, ns)
K (min), mean ± SD	1.17 ± 0.46	1.35 ± 0.21	1.32 ± 0.46 (ns)	1.17 ± 0.31 (ns, ns)	1.02 ± 0.19 (*, a, ns)	1.14 ± 0.52 (ns, ns, ns, ns)
α angle (°), mean ± SD	72.75 ± 5.31	68.98 ± 2.54	70.39 ± 5.77 (ns)	72.15 ± 4.01 (ns, ns)	74.34 ± 2.97 (*, ns, ns)	73.47 ± 5.67 (*, ns, ns, ns)
CK (IU/L), mean ± SD	122.50 ± 181.05	85.92 ± 34.45	88.35 ± 66.00 (ns)	117.15 ± 81.63 (ns, ns)	157.17 ± 98.37 (ns, ns, ns)	132.98 ± 236.92 (ns, ns, ns, ns)
CK-MB (μg/L), mean ± SD	8.3 ± 5.3	5.0 ± 2.8	7.8 ± 3.4 (ns)	9.3 ± 7.3 (*, ns)	7.5 ± 4.5 (ns, ns, ns)	8.7 ± 5.1 (*, ns, ns, ns)
hs-TnI (μg/L), mean ± SD	0.043 ± 0.10	0.005 ± 0.008	0.0057 ± 0.011 (ns)	0.035 ± 0.098 (ns, ns)	0.019 ± 0.019 (ns, ns, ns)	0.062 ± 0.124 (*, a, ns, c)
Mb (μg/L), mean ± SD	237.3 ± 217.6	51.8 ± 41.9	47.0 ± 30.0 (ns)	119.9 ± 115.1 (ns, a)	211.9 ± 168.7 (*, a, ns)	332.9 ± 225.4 (*, a, b, ns)
WBC (10^9^/L), mean ± SD	6.34 ± 2.21	5.55 ± 0.98	7.30 ± 2.96 (*)	6.61 ± 2.26 (ns, ns)	6.47 ± 1.77 (ns, ns, ns)	6.05 ± 2.02 (ns, ns, ns, ns)
CRP (mg/L), mean ± SD	13.8 ± 30.7	12.8 ± 35.7	2.4 ± 3.0 (ns)	8.1 ± 8.4 (ns, a)	17.5 ± 38.9 (ns, ns, ns)	18.9 ± 37.0 (ns, a, ns, ns)
hs-CRP (mg/L), mean ± SD	11.76 ± 31.35	1.58 ± 1.50	2.72 ± 3.69 (ns)	7.56 ± 8.58 (*, ns)	26.24 ± 48.62 (ns, ns, ns)	16.49 ± 41.03 (ns, ns, ns, ns)
SAA (mg/L), mean ± SD	58.1 ± 74.5	94.3 ± 118.3	29.1 ± 41.1 (ns)	51.0 ± 57.6 (ns, ns)	56.2 ± 83.8 (ns, ns, ns)	62.5 ± 78.4 (ns, ns, ns, ns)
LDL (mmol/L), mean ± SD	3.16 ± 1.34	3.01 ± 1.32	3.87 ± 1.92 (ns)	3.56 ± 1.19 (ns, ns)	2.98 ± 0.87 (ns, ns, ns)	2.75 ± 1.03 (ns, a, b, ns)
GLU (mmol/L), mean ± SD	5.37 ± 2.01	5.13 ± 0.71	5.21 ± 1.45 (ns)	5.52 ± 2.11 (ns, ns)	4.95 ± 1.46 (ns, ns, ns)	5.46 ± 2.29 (ns, ns, ns, ns)
SOD (U/mL), mean ± SD	123.44 ± 34.80	163.25 ± 15.98	138.71 ± 44.30 (ns)	115.05 ± 30.44 (*, ns)	114.50 ± 38.61 (ns, ns, ns)	115.06 ± 27.70 (*, ns, ns, ns)

Data are presented as mean ± SD or %, as appropriate. ns, not significant; *, *p* < 0.05 versus the non-CKD group; a, *p* < 0.05 versus CKD stages 1–2; b, *p* < 0.05 versus CKD stage 3; c, *p* < 0.05 versus CKD stage 4 (Dunnett’s T3 test; each CKD stage was compared with all preceding groups). Abbreviations: CKD, chronic kidney disease; CREA, creatinine; eGFR, estimated glomerular filtration rate; UACR, urinary albumin-to-creatinine ratio; sTM, soluble thrombomodulin; TAT, thrombin–antithrombin complex; PIC, plasmin–α2-plasmin inhibitor complex; tPAIC, tissue plasminogen activator–plasminogen activator inhibitor-1 complex; PT, prothrombin time; APTT, activated partial thromboplastin time; FIB, fibrinogen; FDP, fibrin(ogen) degradation products; R, reaction time; MA, maximum amplitude; K, clot formation time; α angle, alpha angle; CK, creatine kinase; CK-MB, creatine kinase-MB; hs-TnI, high-sensitivity cardiac troponin I; Mb, myoglobin; WBC, white blood cell count; CRP, C-reactive protein; hs-CRP, high-sensitivity C-reactive protein; SAA, serum amyloid A; LDL, low-density lipoprotein; GLU, glucose; SOD, superoxide dismutase.

## Data Availability

The public transcriptomic datasets analyzed in this study are available in the NCBI Gene Expression Omnibus under accession numbers GSE217650, GSE281539 and GSE183276. Kidney Precision Medicine Project (KPMP) data are available through the relevant KPMP data portal, subject to its access conditions. The de-identified clinical data supporting the findings of this study are available from the corresponding author upon reasonable request, subject to institutional approval and applicable privacy regulations.

## Abbreviations

The following abbreviations are used in this manuscript:

CKD	Chronic kidney disease
TM	Thrombomodulin
sTM	Soluble thrombomodulin
THBD	Thrombomodulin gene
eGFR	Estimated glomerular filtration rate
UACR	Urinary albumin-to-creatinine ratio
TEG	Thromboelastography
PT	Prothrombin time
APTT	Activated partial thromboplastin time
FDP	Fibrin degradation products
Mb	Myoglobin
CK	Creatine kinase
CK-MB	Creatine kinase-MB
hs-TnI	High-sensitivity cardiac troponin I

## Appendix A

**Table A1:** Receiver operating characteristic (ROC) analyses of sTM, eGFR, and UACR for discriminating CKD, with DeLong test comparisons against sTM.

Comparison	Marker	AUC (95% CI)	Cutoff	Sensitivity	Specificity	DeLong vs. sTM
CKD all vs. non-CKD	sTM	0.971 (0.952–0.989)	10.0	92.7%	100.0%	—
CKD all vs. non-CKD	eGFR	0.920 (0.880–0.959)	45.5	75.5%	100.0%	*p* = 0.0084
CKD all vs. non-CKD	UACR	0.872 (0.787–0.958)	70.2	80.6%	87.5%	*p* = 0.3726
Early CKD vs. non-CKD	sTM	0.854 (0.762–0.947)	10.0	63.4%	100.0%	—
Early CKD vs. non-CKD	eGFR	0.565 (0.403–0.727)	87.5	53.7%	77.8%	*p* = 0.0012
Early CKD vs. non-CKD	UACR	0.815 (0.674–0.955)	75.8	72.4%	87.5%	*p* = 0.7082
Advanced CKD vs. others	sTM	0.967 (0.940–0.995)	18.4	96.6%	86.7%	—
Advanced CKD vs. others	eGFR	1.000 (1.000–1.000)	34.3	100.0%	100.0%	*p* = 0.0272
Advanced CKD vs. others	UACR	0.781 (0.675–0.888)	409	88.6%	59.5%	*p* < 0.001

Optimal cutoffs were determined by the Youden index. For eGFR, the sign was reversed and values below the cutoff were considered test-positive. “DeLong vs. sTM” denotes *p* values from DeLong tests comparing the AUC of each marker with that of sTM. Early CKD, CKD stages 1–2; advanced CKD, CKD stages 4–5 versus non-CKD and stages 1–2 combined. Units of cutoffs: sTM, TU/mL; eGFR, mL/min/1.73 m^2^; UACR, mg/g. AUC, area under the curve; CI, confidence interval; CKD, chronic kidney disease; eGFR, estimated glomerular filtration rate; sTM, soluble thrombomodulin; UACR, urinary albumin-to-creatinine ratio.

**Table A2:** Sensitivity analysis of circulating sTM concentrations across CKD stages after excluding patients receiving dialysis.

CKD Stage	*n*	sTM (TU/mL), Mean ± SD	sTM, Median (IQR)
non-CKD	19	6.68 ± 1.96	6.90 (5.35–7.92)
CKD1-2	41	13.65 ± 7.35	11.70 (8.30–15.60)
CKD3	44	18.88 ± 7.97	17.85 (14.50–20.52)
CKD4	23	24.90 ± 9.00	21.80 (19.10–29.10)
CKD5	22	33.21 ± 6.35	32.35 (28.88–38.85)

Data are *n* or sTM concentration (mean ± SD; median [IQR]). Overall comparison across CKD stages was performed using the Kruskal–Wallis test (*H* = 87.9, *p* < 0.0001). CKD, chronic kidney disease; IQR, interquartile range; sTM, soluble thrombomodulin.

**Table A3:** Multivariable linear regression of log-transformed (natural logarithm) circulating sTM.

Model	Variable	*β*	SE	95% CI	*p*	*n*/adj *R*^2^
M1	Intercept	3.6107	0.0321	3.5474 to 3.6740	<0.001	263/0.634
	eGFR	−0.0139	0.0007	−0.0152 to −0.0126	<0.001	
M2	Intercept	4.1372	0.1194	3.9021 to 4.3724	<0.001	263/0.686
	eGFR	−0.0147	0.0006	−0.0160 to −0.0135	<0.001	
	Age	−0.0093	0.0017	−0.0127 to −0.0060	<0.001	
	Male sex	0.1441	0.0441	0.0573 to 0.2309	0.001	
M3	Intercept	3.7004	0.2951	3.1147 to 4.2861	<0.001	105/0.673
	eGFR	−0.0116	0.0013	−0.0141 to −0.0090	<0.001	
	Age	−0.0110	0.0029	−0.0168 to −0.0052	<0.001	
	Male sex	0.1256	0.0645	−0.0025 to 0.2537	0.055	
	Hypertension	−0.1277	0.0846	−0.2957 to 0.0403	0.135	
	Diabetes mellitus	−0.0423	0.0676	−0.1766 to 0.0920	0.533	
	Heart failure	0.0131	0.0918	−0.1691 to 0.1953	0.887	
	Dialysis	0.0139	0.1345	−0.2530 to 0.2808	0.918	
	Log (UACR)	0.0773	0.0180	0.0416 to 0.1130	<0.001	
M4	Intercept	4.0816	0.1860	3.7145 to 4.4488	<0.001	172/0.678
	eGFR	−0.0135	0.0012	−0.0159 to −0.0112	<0.001	
	Age	−0.0096	0.0023	−0.0141 to −0.0052	<0.001	
	Male sex	0.1816	0.0581	0.0669 to 0.2964	0.002	
	Hypertension	−0.0746	0.0770	−0.2266 to 0.0774	0.334	
	Diabetes mellitus	−0.0078	0.0598	−0.1259 to 0.1103	0.896	
	Heart failure	0.0317	0.0689	−0.1043 to 0.1676	0.646	
	Dialysis	0.1470	0.0797	−0.0104 to 0.3044	0.067	
	Log (CRP)	0.0002	0.0187	−0.0368 to 0.0372	0.992	

*β*, unstandardized regression coefficient; SE, standard error; CI, confidence interval; *n*, complete-case sample size; adj R^2^, adjusted coefficient of determination. Models: M1, eGFR only; M2, M1 + age + sex; M3, M2 + hypertension + diabetes mellitus + heart failure + dialysis + log-transformed UACR; M4, M2 + hypertension + diabetes mellitus + heart failure + dialysis + log-transformed C-reactive protein (see Section 2.5). eGFR is expressed per 1 mL/min/1.73 m^2^; log-transformed UACR and log-transformed CRP are per 1 log-unit.

**Table A4:** Pearson correlation analyses with Benjamini–Hochberg FDR correction.

Parameter Group	Variable	*n*	Pearson *r*	*p*	*q* (BH-FDR)
Renal	CREA	263	0.7398	<0.0001	<0.0001
	Urea	263	0.6033	<0.0001	<0.0001
	eGFR	263	−0.7122	<0.0001	<0.0001
	UACR	106	0.4963	<0.0001	<0.0001
Thromboelastography (TEG)	R	208	−0.0437	0.531	0.559
	K	208	−0.1811	0.0088	0.015
	α angle	208	0.2727	<0.0001	0.0002
	MA	208	0.2429	0.0004	0.0010
Coagulation/fibrinolysis	PT	225	0.1936	0.0036	0.0071
	APTT	224	0.2003	0.0026	0.0058
	FIB	226	0.2777	<0.0001	<0.0001
	D-dimer	180	0.1668	0.025	0.036
	FDP	112	0.2157	0.022	0.034
Cardiac	Mb	171	0.5702	<0.0001	<0.0001
	CK	235	0.1162	0.075	0.089
	CK-MB	249	0.1298	0.041	0.054
	hs-TnI	172	0.2041	0.0072	0.013
Coagulation–fibrinolysis complexes	TAT	278	−0.0459	0.446	0.495
	PIC	278	0.0273	0.651	0.651
	tPAIC	278	−0.1088	0.070	0.088

Benjamini–Hochberg false discovery rate (FDR) correction was applied across all 20 correlation tests; *q* < 0.05 was considered statistically significant. *n* indicates pairwise complete-case sample size. Abbreviations are as defined in Table 1.
